# A new sweetpotato variety ‘Michishizuku’ for alcohol and starch production with high yield and resistance to foot rot

**DOI:** 10.1270/jsbbs.25055

**Published:** 2026-04-01

**Authors:** Akira Kobayashi, Yukari Kawata, Takeo Sakaigaichi, Keisuke Suematsu, Yumi Kai, Tetsufumi Sakai, Yasuhiro Takahata

**Affiliations:** 1 Kyushu Okinawa Agricultural Research Center, National Agriculture and Food Research Organization, 6651-2 Yokoichi, Miyakonojo, Miyazaki 885-0091, Japan

**Keywords:** sweetpotato, variety, alcohol, *shochu*, starch, foot rot disease

## Abstract

We developed a new sweetpotato variety, ‘Michishizuku’, as the raw material for *imo-shochu*, a traditional Japanese distilled liquor made from sweetpotaoes, and for starch production. Michishizuku was selected from a cross between ‘Konaishin’ and Kyukei 09187-14 and was released in 2021. Its total yield was higher than that of ‘Koganesengan’, slightly higher than that of ‘Shiroyutaka’, and slightly lower than that of Konaishin. The starch content of Michishizuku was significantly higher than those of the other varieties, and its starch yield was 146–147% that of Koganesengan and 93–102% that of Konaishin. *Shochu* brewing tests confirmed that the alcohol yield of Michishizuku was higher than that of Koganesengan. In addition, the flavor of *shochu* made from Michishizuku (Michishizuku *shochu*) was similar to that of Koganesengan *shochu*, and its sensory evaluation score was slightly higher than that of Koganesengan *shochu*. Based on these results, Michishizuku is highly suitable for *shochu* brewing. The viscosity characteristics of the starch of Michishizuku were similar to those of Shiroyutaka and Konaishin, and its whiteness was as high as that of Shiroyutaka. Therefore, Michishizuku is also suitable as a raw material for starch production. Michishizuku exhibited moderately strong resistance to foot rot, which causes severe damage to sweetpotato in the Southern Kyushu area. These characteristics suggest that Michishizuku will contribute to reductions in damage caused by foot rot and the stable production of *shochu* and starch.

## Introduction

*Imo-shochu* is a Japanese traditional distilled liquor made from sweetpotato (*Ipomoea batatas* (L.) Lam.) and is popular with consumers in Japan. The *shochu* industry is thriving in southern Kyushu, and sweetpotatoes as the raw material for *shochu* production are an important crop in agricultural production and the local economy in this region. The leading variety used for *shochu* production is ‘Koganesengan’, developed at Kyushu National Agricultural Experiment Station in 1966 (Sakai *et al.* 1967), and it was the second most cultivated variety in Japan in terms of its cultivated area after ‘Beniharuka’ (Kai *et al.* 2017) in 2022 (MAFF 2024). Koganesengan exhibits high yield, wide regional adaptability, and is used as a raw material not only for *shochu*, but also food processing, such as chips, *kenpi*, and paste. *Shochu* made from Koganesengan has an excellent flavor of sweetpotato and is superior in terms of the balance between flavor and sweetness. Despite the shortcomings of Koganesengan, such as deep grooves on the storage root caused by a physiological disorder, its poor external shape, and its susceptibilities to a southern root-knot nematode (*Meloidogyne incognita*), coffee root-lesion nematode (*Pratylenchus coffeae*), black rot (*Ceratocystis fimbriata*), soil rot (*Streptomyces ipomoeae* (Person and Martin) Waksman and Henrici.), and fusarium wilt (*Fusarium oxysporum*), it is still regarded as the most suitable variety for *shochu* and has been used for decades as the leading variety.

However, since the first case of foot rot disease in Japan in 2018 (Kobayashi 2019), Koganesengan has suffered extensive damage, such as vine death and storage root deterioration, and the cultivation area of Koganesengan in southern Kyushu has decreased from 7,878 ha in 2018 to 6,585 ha in 2022 (MAFF 2021, 2024), which has severely hindered its stable supply as the raw material for *shochu*. Foot rot is a soil-borne disease caused by the fungus *Diaporthe destruens* (Harter) Hirooka, Minosh., and Rossman that is difficult to control. Therefore, the development of a sweetpotato variety that is resistant to foot rot and may be used as a raw material for *shochu* instead of Koganesengan with the similar characteristic flavor of *shochu* made from Koganesengan is awaited.

In the present study, we developed a new sweetpotato variety with high yield, resistance to foot rot, and suitability as a raw material for *shochu* and starch production.

## Materials and Methods

### Breeding process

**Fig. 1.**
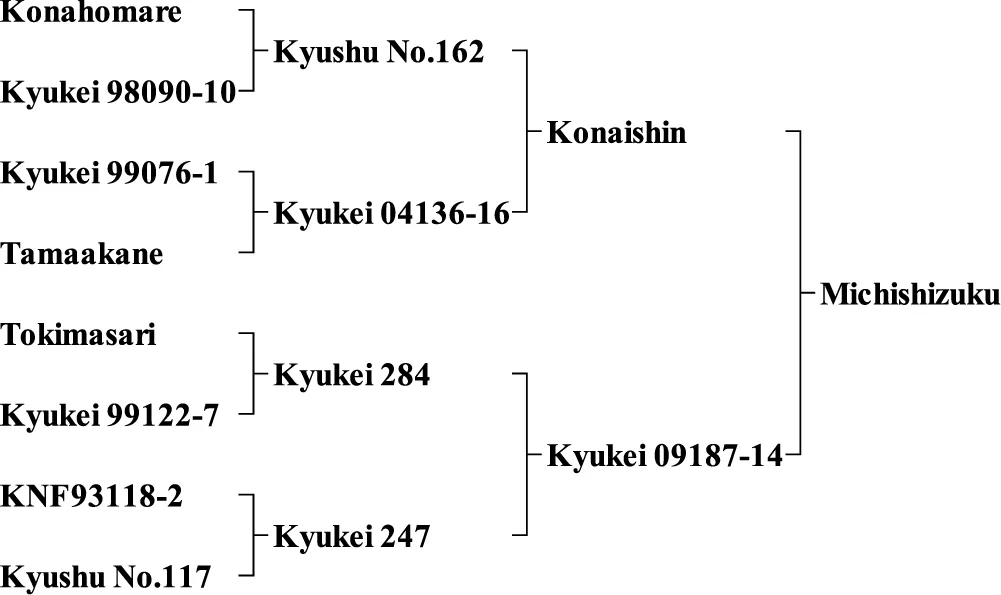
Pedigree of Michishizuku.

The new variety ‘Michishizuku’ was initially termed Kyukei 14195-7, renamed Kyukei 359, and then finally called Kyushu No. 200. This clone was selected from the progenies of a cross between ‘Konaishin’ (Kobayashi *et al.* 2024), as the female parent, and Kyukei 09187-14, as the male parent. A complete pedigree is shown in Fig. 1. Konaishin is a variety for starch production with high yield and resistance to multiple diseases and pests, including foot rot. Kyukei 09187-14 is a high-yield breeding line with a high dry matter content. When steamed, its storage roots exhibit a powdery texture and good eating quality. In 2015, 370 seeds were sown in a greenhouse, and stem cuttings from each seedling were then transplanted in a field at the Kyushu Okinawa Agricultural Research Center, NARO, Miyakonojo, Miyazaki, Japan (31°45 N, 131°00 E). Based on the size, shape, and number of storage roots, 34 clones were visually selected at harvest. In the following 2 years (2016–2017), the selected clones were propagated from storage roots and further evaluated for agricultural characteristics. A preliminary yield trial was conducted in 2018 using 12.6 m^2^ plots with two replicates, followed by 3-year yield trials from 2019 to 2021 using 12.6 m^2^ plots with three replicates. The clonal line selected in 2018 was designated as Kyukei 359 and renamed in 2020 as Kyushu No. 200. Local adaptability was evaluated at the two different locations, Miyazaki and Kagoshima prefectures, in 2019–2020. Performance tests for the recommended varieties were conducted at Miyazaki and Kagoshima prefectures in 2021, and then at 3 farm sites in Miyazaki and Kagoshima prefectures in 2020–2021. The suitability for *shochu* brewing was evaluated by members of the research group for the processing quality of sweetpotato supported by the Japan Root and Tuber Crops Development Association Inc., Kirishima Shuzo Co., Ltd. (Miyazaki, Japan) for 3 years (2019–2021), and Okuchi Shuzo Co., Ltd. (Kagoshima, Japan) in 2019. Kyushu No. 200 was released as Michishizuku in 2021, and was officially registered by the Ministry of Agriculture, Forestry and Fisheries of Japan in 2024.

### Agronomic characteristics

Morphological traits were evaluated according to the test guidelines of The International Union for the Protection of New Varieties of Plants. The agronomic traits of Michishizuku, Koganesengan, ‘Shiroyutaka’ (Sakamoto *et al.* 1987), and Konaishin under standard and long-term cultivations were examined in 2019–2022 and 2019–2021, respectively. Shiroyutaka is a main variety for starch production, while Konaishin is a variety for starch production with high yield and resistance to multiple diseases and pests registered in 2022 (Kobayashi *et al.* 2024). An analysis of variance (ANOVA) of total yield, dry matter content, starch content, starch yield, average size of the storage root, and number of storage roots per hill in standard cultivation was performed. Tukey’s HSD test was used to identify significant differences among varieties at *P* < 0.05. All analyses were performed in JMP 12 (SAS Institute).

Resistance tests for foot rot disease were conducted in a field infested with foot rot in 2021–2022 according to the method performed by Kobayashi *et al.* (2025). Resistance tests for a southern root-knot nematode and coffee root-lesion nematode were conducted in fields infested with these nematodes in the Miyakonojo station of KARC/NARO in 2018, 2019, and 2021. The tests for the southern root-knot nematode were carried out in fields where *M. incognita*, race SP1 was the predominant population, while those for the coffee root-lesion nematode were conducted in fields infested with *P. coffeae*. Resistance tests for soil rot and black rot disease were conducted by Nagasaki and Tokushima prefectures in 2019, respectively. Resistance test for fusarium wilt (*F. oxysporum*) was performed using the pot test according to the method developed by Yamakawa *et al.* (2022) in 2025.

Laboratory-scale *shochu* production was conducted by Kirishima Shuzo Co., Ltd. using a mash composition consisting of 0.5 kg of koji rice, 2.5 kg of sweetpotatoes, and 2010 ml of water. Distillation was performed under atomospheric pressure using a small glass distiller. In a separate trial conducted by Okuchi Shuzo Co., Ltd., *shochu* was producted using 1.0 kg of koji rice, 5.0 kg of sweetpotatoes, and 3900 ml of water, and distilled under atmospheric pressure using a small stainless-steel distiller. The alcohol concentration of the secondary mash, the main fermentation stage in *shochu* production, was measured using the official analytical method prescribed by the National Tax Agency of Japan. In the sensory evaluation, the taste, aroma, and overall flavor balance of the *shochu* were comprehensively assessed.

## Results

### Morphological description

The growth habit of Michishizuku was ‘spreading’. Anthocyanin coloration of the nodes and internodes of Michishizuku was classified as ‘absent or very weak’. The leaves of Michishizuku had three very shallow lobes in the leaf blade. The color on both the upper and lower sides of Michishizuku leaves was green. The extent and intensity of anthocyanin coloration on abaxial veins on the lower side of the leaf blade were classified as ‘absent or very small’ and ‘very weak’, respectively. Anthocyanin coloration of the nectary of the leaf blade was absent.

**Fig. 2.**
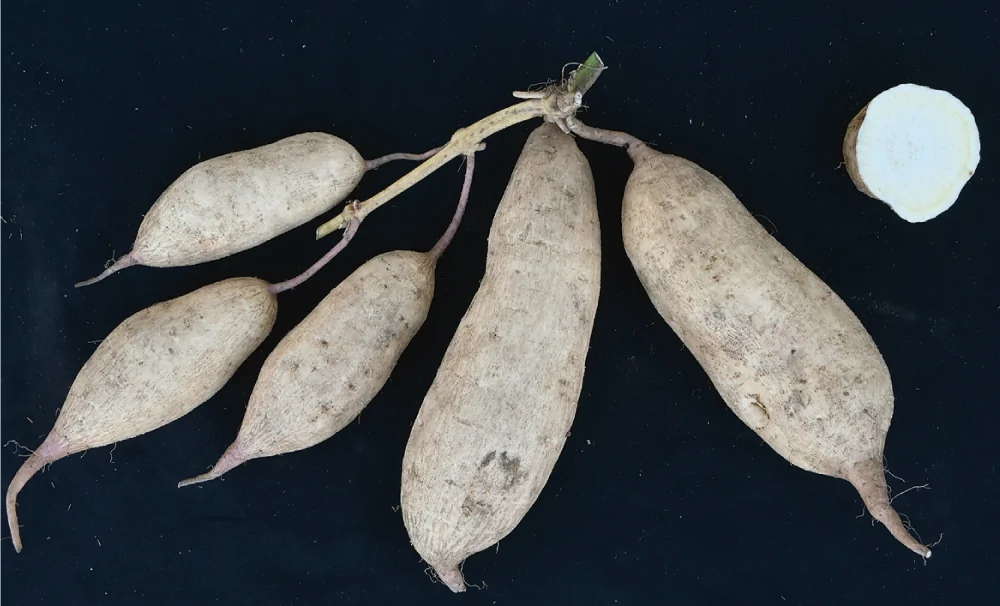
Storage roots of Michishizuku.

The storage root shape of Michishizuku was elliptic and the flesh color was light beige (Fig. 2). Michishizuku had particolored pink/light beige skin storage roots, namely, the skin color around both ends of the storage roots was pink and the remainder was light beige. The storage root eyes were shallow.

### Agronomic characteristics

ANOVA of total yield, dry matter content, starch content, starch yield, average size of the storage root, and number of storage roots per hill in standard cultivation was performed (Table 1). All the parameters were significantly different among the varieties and in different years. In addition, there were significant year by variety interactions in all the parameters.

The total yield of Michishizuku in standard cultivation was 429 kg/a on average for 4 years, which was significantly higher than that of Koganesengan (341 kg/a), higher than that of Shiroyutaka (378 kg/a), and lower than that of Konaishin (468 kg/a) (Table 2). The total yield of Michishizuku in long-term cultivation was 553 kg/a on average for 3 years, which was higher than those of Koganesengan (457 kg/a) and Shiroyutaka (484 kg/a) and lower than that of Konaishin (668 kg/a) (Table 2). The average size of the storage root in standard cultivation was 237 g, which was similar to those of Shiroyutaka (253 g) and Konaishin (255 g), and significantly bigger than that of Koganesengan (209 g). The average size of the storage root in long-term cultivation was 295 g, which was similar to those of Koganesengan (287 g) and Shiroyutaka (333 g), and significantly smaller than that of Konaishin (410 g). The number of storage roots per hill in standard cultivation was 4.9, which was similar to those of Konaishin (5.0) and Koganesengan (4.5), and more than that of Shiroyutaka (4.1). The number of storage roots per hill in long-term cultivation was 6.4, which was similar to those of Koganesengan (5.5) and Konaishin (5.6), and significantly more than that of Shiroyutaka (5.2). The dry matter contents of Michishizuku in standard and long-term cultivations were 40.0 and 41.0%, respectively, which were significantly higher than those of Koganesengan (35.8 and 35.4%), Shiroyutaka (37.3 and 36.3%), and Konaishin (37.4 and 37.5%). Similarly, the starch contents of Michishizuku in standard and long-term cultivations were 28.6 and 30.4%, respectively, which were significantly higher than those of the other varieties. The starch yield of Michishizuku in standard cultivation was 123 kg/a, which was significantly higher than those of Koganesengan (84 kg/a) and Shiroyutaka (99 kg/a) and similar to that of Konaishin (121 kg/a). Similarly, the starch yield of Michishizuku in long-term cultivation was 168 kg/a, which was significantly higher than those of Koganesengan (114 kg/a) and Shiroyutaka (126 kg/a) and similar to that of Konaishin (180 kg/a).

In terms of physiological traits in the storage roots, the deep grooves shown in Koganesengan were rare in Michishizuku. The few skin veins, which appear as blood vessel-like patterns on the surface of storage roots, were slightly observed in Konaishin, but were absent in Michishizuku as in Koganesengan and Shiroyutaka. Michishizuku was slightly more susceptible to storage root crack than Shiroyutaka, and more susceptible than Koganesengan and Konaishin.

An evaluation of the suitability of Michishizuku for *shochu* brewing revealed that both the alcohol content of the second mash (the main fermentation stage in *shochu* production) and the yield of alcohol were higher than those of Koganesengan (Table 3). The average sensory evaluation score of *shochu* made from Michishizuku (Michishizuku *shochu*) was slightly higher than that of Koganesengan (Koganesengan *shochu*) and the number of evaluators who had a positive impression was also higher for Michishizuku *shochu* than for Koganesengan *shochu*. In addition, Michishizuku *shochu* exhibited flavor attributes such as sweetness, sweet aroma, raw material-derived notes, and ester-like notes, which are sensory features similar to those observed in Koganesengan *shochu*. Approximately half of the evaluators in 2020 and about 70% in 2021 rated the flavor profile of Michishizuku *shochu* as similar to that of Koganesengan *shochu*, suggesting that Michishizuku *shochu* exhibits comparable flavor characteristics and quality. Based on these results, Michishizuku is considered a suitable raw material for *shochu* production.

The starch pasting temperature of Michishizuku was the same as that of Koganesengan and slightly lower than those of Shiroyutaka and Konaishin as assessed using a Rapid Visco Analyzer (Table 4). Significant differences in peak viscosity and breakdown were not observed among the four varieties. The setback of starch of Michishizuku was not significantly different from those of the three other varieties. The whiteness of starch of Michishizuku was the same as that of Shiroyutaka, slightly higher than that of Konaishin, and higher than that of Koganesengan.

### Disease evaluations

Michishizuku exhibited moderately strong resistance to foot rot (Table 5). In the field infested with foot rot, the incidence of disease was markedly lower than that on Koganesengan, but was not significantly different from that on the moderately strong resistant varieties Konaishin and ‘Benimasari’ (Ishiguro *et al.* 2004). The yield of the marketable storage roots of Michishizuku was significantly higher than that of Koganesengan, lower than that of Konaishin in 2021, and similar to that of Konaishin in 2022. The ratio of the marketable storage roots of Michishizuku was 51% in 2021 and 83% in 2022, which did not significantly differ from that of Konaishin. Michishizuku exhibited stronger resistance to southern root-knot nematode race SP1, which is the predominant race of *M*. *incognita* in Kumamoto prefecture, than Koganesengan, which was rated as susceptible, and similar resistance to Konaishin and Shiroyutaka. Michishizuku exhibited moderately strong resistance to the coffee root-lesion nematode and soil rot. The resistance class of Michishizuku to black rot and fusarium wilt were classified as “medium”.

## Discussion

Michishizuku is a variety developed to replace Koganesengan as a raw material for *shochu*. It is high yielding, has high starch content, is resistant to foot rot, and is also sufficiently versatile to be used as a starch material.

The total yields of Michishizuku in standard and long-term cultivations were 126 and 121% those of Koganesengan, respectively (Table 2). In comparisons with varieties for starch material, the total yield of Michishizuku in standard cultivation was 113% that of Shiroyutaka and 92% that of Konaishin, while in long-term cultivation, it was 114% that of Shiroyutaka and 83% that of Konaishin. Therefore, the yield of Michishizuku was higher than that of Koganesengan, slightly higher than that of Shiroyutaka, and slightly lower than that of Konaishin. However, since the starch content in the storage root of Michishizuku was 4.1 to 5.4 points higher than that in Koganesengan, 2.4 to 4.3 points higher than that in Shiroyutaka, and 2.8 to 3.4 points higher than that in Konaishin, the starch yield of Michishizuku was 146 to 147% that of Koganesengan, 124 to 133% that of Shiroyutaka, and 93 to 102% that of Konaishin. No significant differences were observed in starch yield between Michishizuku and Konaishin, demonstrating that Michishizuku is a high-yielding variety not only for *shochu* material, but also for starch material. Since the alcohol present in *shochu* is made from starch, a high starch content is directly linked not only to the starch yield, which is important as a raw material for starch, but also to the yield of alcohol in *shochu* brewing, which is also important as a raw material for *shochu*. *Shochu* brewing tests confirmed that the alcohol content of the secondary mash of Michishizuku was 0.7 to 1.8 points higher than that of Koganesengan, and the alcohol yield of Michishizuku was 105 to 112% that of Koganesengan (Table 3).

A previous study revealed that the flavors of *shochu* varied depending on the sweetpotato variety used as the raw material for brewing (Kamiwatari *et al.* 2006). Koganesengan *Shochu* exhibits a distinctive flavor profile that is mellow and smooth, free from off-flavors, and most notably characterized by a sweet aroma and sweetness strongly reminiscent of steamed sweetpotatoes, which are considered the defining attributes of its flavor. This type of *shochu* is highly valued by consumers. Furthermore, Koganesengan is a high-yielding variety with a high alcohol yield and is easy to procure. Consequently, it has been used as the main raw material for *shochu* production over many years. ‘Joy White’ (Yamakawa *et al.* 1995), ‘Tokimasari’ (Katayama *et al.* 2009), ‘Tamaakane’ (Sakai *et al.* 2011), ‘Satsumamasari’ (Katayama *et al.* 2013), ‘Koganemasari’ (Sakai *et al.* 2013), and other varieties have been developed as raw materials for *shochu*. However, the flavors of *shochu* made from these varieties differ from that of Koganesengan *shochu*. Although they have contributed to diversifying the flavor of *shochu*, they have not become a substitute for Koganesengan. Therefore, alternative varieties to Koganesengan are required not only for cost advantages, such as higher alcohol yields and the easier handling of raw materials, but also to achieve a similar flavor to that of Koganesengan *shochu*. In a sensory evaluation of *shochu*, Michishizuku *shochu* exhibited flavor attributes such as sweetness, sweet aroma, and raw material-derived notes, which were similar to those observed in Koganesengan *shochu*. Sensory evaluations conducted in 2020 and 2021 further supported this similarity; more than half of the evaluators rated the flavor profile of Michishizuku *shochu* as similar to that of Koganesengan *shochu* (Table 3).

Yamamoto *et al.* (2021) evaluated the brewing characteristics of *shochu* by analyzing the concentrations of characteristic flavor compounds, including linalool, α-terpineol, citronellol, nerol, geraniol, and β-damascenone, in *shochu* made from Michishizuku and Koganesengan. Although the concentration of β-damascenone, a compound contributing to a sweet aroma, was slightly higher in Michishizuku *shochu*, the levels of the other characteristic flavor compounds were nearly the same in both varieties of *shochu*, indicating that there were no substantial differences in their flavor compound profiles. Taken together, these findings suggest that Michishizuku *shochu* has a flavor profile that resembles that of Koganesengan *shochu*, despite exhibiting slightly enhanced sweetness. Therefore, Michishizuku can be regarded as highly suitable for shochu production in terms of its flavor characteristics.

Koganesengan has some disadvantages, the most serious of which is its susceptibility to foot rot. Its resistance class is rated “moderately weak”. The level of resistance of Michishizuku to foot rot was slightly lower than that of Konaishin, but was classified as “moderately strong”, the same class as Konaishin (Table 5). In the resistance test, the disease incidence of Michishizuku was significantly lower than that of Koganesengan and similar to that of the moderately strong resistant varieties Konaishin and Benimasari. In addition, the yield of the apparently healthy storage roots of Koganesengan was 24 kg/a in 2021 and 125 kg/a in 2022, while that of Michishizuku was 147 kg/a in 2021 and 389 kg/a in 2022, which was approximately 3- to 6-fold higher than that of Koganesengan. Control measures for foot rot include practices such as using virus-free seedlings, disinfecting seedlings, and applying chemical treatments. Among these measures, the use of resistant varieties is considered an effective and cost-efficient measure. Michishizuku is expected to contribute to reducing damage caused by foot rot as the first resistant variety capable of replacing Koganesengan.

In addition, while Koganesengan was susceptible to major sweetpotato diseases and pests, Michishizuku was resistant to the southern root-knot nematode, coffee root-lesion nematode, and soil rot, and exhibited moderate resistance to fusarium wilt and black spot disease, suggesting its potential for stable production. Furthermore, the shape of the storage root of Michishizuku is smooth and elliptical without deep grooves, which is superior to that of Koganesengan.

The viscosity characteristics of the starch of Michishizuku were similar to those of Shiroyutaka and Konaishin, and the whiteness of its starch was as high as that of Shiroyutaka (Table 4). Therefore, Michishizuku is also suitable as a raw material for starch. The cultivation of Konaishin has been expanding, with a cultivation area of 1,631 ha in 2022, exceeding that of Shiroyutaka at 1,592 ha. However, Konaishin has the disadvantage of the root stalk connecting the storage root to the stem being very strong, which makes it difficult and time-consuming to separate storage roots from the stem at harvest. The strength of the root stalk of Michishizuku was found to be weaker than that of Konaishin and similar to that of Koganesengan (data not shown); therefore, it may be easier to separate the storage roots of Michishizuku at harvest. These results indicate that it may be preferable to select Michishizuku or Konaishin as the variety for starch material, taking into account factors such as yield, regional adaptability, resistance to diseases and pests, and work efficiency during harvesting.

In southern Kyushu, the damage caused by foot rot has decreased due to efforts to control the disease. One of the management strategies employed involves the use of resistant varieties, which is expected to contribute to reductions in damage caused by foot rot, and the stable production of *shochu* and starch by expanding Michishizuku instead of Koganesengan in areas where varieties for raw materials for *shochu* are produced and together with Konaishin in areas where varieties for starch material are produced.

## Author Contribution Statement

AK, YK, TS, KS, YK, TS, and YT contributed to the breeding of Michishizuku. AK drafted the manuscript, and all authors approved the final manuscript.

## Figures and Tables

**Table 1.** Analysis of variance of several parameters among four sweetpotato varieties in a four-year trial of standard cultivation

Parameter	Sources	d.f.	Sum of square	*F*-value
Total yield	Variety	3	112611.11	43.4121***
	Year	3	36082.40	13.9099***
	Year × variety	9	37760.74	4.8523***
	Error	32	27669.35	
	Total	47	214123.61	
Dry matter content	Variety	3	104.28	22.9543***
	Year	3	92.86	20.439***
	Year × variety	9	30.37	2.2283**
	Error	32	48.46	
	Total	47	275.97	
Starch content	Variety	3	105.31	72.2015***
	Year	3	29.20	20.0217***
	Year × variety	9	15.96	3.6469**
	Error	32	15.56	
	Total	47	166.04	
Starch yield	Variety	3	12587.35	73.5600***
	Year	3	3384.04	19.7762***
	Year × variety	9	2747.47	5.3520***
	Error	32	1825.25	
	Total	47	20544.10	
Average size of the storage root	Variety	3	16364.73	10.7764***
	Year	3	67010.12	44.1273***
	Year × variety	9	11158.28	2.4493*
	Error	32	16198.03	
	Total	47	110731.16	
No. of storage roots per hill	Variety	3	5.95	10.0142***
	Year	3	18.70	31.458***
	Year × variety	9	7.07	3.9616**
	Error	32	6.34	
	Total	47	38.06	

*, **, *** Significant at the 5%, 1%, and 0.1% levels, respectively.

**Table 2.** Comparative performance of Michishizuku and control varieties

Variety	Cropping type	Total yield*^c^* (kg/a)	Average size of the storage root (g)	No. of storage roots per hill	Dry matter content (%)	Starch content (%)	Starch yield*^d^* (kg/a)
Michishizuku	standard*^a^*	429 ab	237 a	4.9 a	40.0 a	28.6 a	123 a
Koganesengan		341 c	209 b	4.5 ab	35.8 c	24.5 c	84 c
Shiroyutaka		378 bc	253 a	4.1 b	37.3 b	26.2 b	99 b
Konaishin		468 a	255 a	5.0 a	37.4 b	25.8 b	121 a
Michishizuku	long-term*^b^*	553 ab	295 b	6.4 a	41.0 a	30.4 a	168 a
Koganesengan		457 b	287 b	5.5 ab	35.4 b	25.0 c	114 b
Shiroyutaka		484 b	333 b	5.2 b	36.3 b	26.1 bc	126 b
Konaishin		668 a	410 a	5.6 ab	37.5 b	27.0 b	180 a

Values within a column followed by different letters are significantly different according to Tukey’s test (*P* = 0.05). *^a^* The planting dates were May 8, 2019, April 23, 2020, May 10, 2021, and May 16, 2022. The harvest dates were October 8, 2019, October 14, 2020, October 19, 2021, and October 5, 2022. Black biodegradable film was mulched. Yield data recorded from a single-row plot 4.2 m long with 35-cm plant spacing and 4 replications. Average of 3 sites each year. *^b^* The planting dates were April 23, 2019, April 17, 2020, and April 19, 2021. The harvest dates were November 5, 2019, November 4, 2020, and November 16, 2021. Transparent plastic film (2019–2020) and black film (2021) were mulched. Yield data recorded from a single-row plot 4.5 m long with 45-cm plant spacing and 4 replications. Average of 2 sites each year. *^c^* Total yield includes storage roots >50 g. *^d^* Starch yield was calculated from the total yield and starch content.

**Table 3.** *Shochu* brewing tests and sensory evaluation of *shochu* (2019–2021)

Year	Variety	No. of evaluators	Alcohol content of the second mash (%)	Yield of alcohol (L/ton for the storage root)	Evaluation score*^a^*	No. of evaluators with a positive impression	Similarity with KS *shochu^b^*	Sensory evaluation comments*^c^*
Kirishima Shuzo								
2019	Michishizuku	20	16.9	239	3.4	6	–	raw material-derived notes, sweet aroma, ester notes, sweetness
	Koganesengan		16.0	221	3.2	4	–	raw material-derived notes, sweet aroma, sweetness
2020	Michishizuku	23	16.7	242	3.7	10	12	sweetness (17), sweet aroma (15), raw material-derived notes (10)
	Koganesengan		15.3	220	3.0	6	–	raw material-derived notes (17), sweetness (17), sweet aroma (15)
2021	Michishizuku	15	16.9	243	3.8	8	10	sweet aroma (13), sweetness (12), raw material-derived notes (9)
	Koganesengan		15.1	217	3.2	5	–	sweet aroma (12), sweetness (12), raw material-derived notes (10)
Okuchi Shuzo								
2019	Michishizuku	15.4	201	–	–	–		
	Koganesengan	14.7	191	–	–	–		

*Shochu* brewing tests and tasting were conducted by Kirishima Shuzo Co., Ltd. (2019–2021) and Okuchi Shuzo Co., Ltd. (2019). *^a^* Evaluation scores were averaged by the assessors using a five-point scale (1–5). The higher the number, the higher the rating. *^b^* Similarity with KS (Koganesengan) *shochu* indicates the number of evaluators who rated the quality of the *shochu* (aroma and flavor) as similar to that of Koganesengan *shochu*. *^c^* The number in parentheses indicates the number of evaluators.

**Table 4.** Pasting properties and whiteness of sweetpotato starch (Means of 2019–2022)

Variety	Pasting temperature (℃)	Peak temperature (℃)	Peak viscosity (RVU)	Breakdown (RVU)	Setback (RVU)	Whiteness*^a^*
Michishizuku	74.7 b	82.2 bc	199.7 a	96.2 a	122.4 ab	91.2 a
Koganesengan	74.9 b	81.6 c	193.7 a	87.4 a	118.4 b	88.3 b
Shiroyutaka	76.2 a	83.7 ab	206.9 a	91.1 a	128.3 a	90.6 a
Konaishin	76.7 a	84.3 a	200.5 a	88.1 a	121.0 ab	90.3 ab

Starch samples were prepared from storage roots grown by standard mulching cultivation. The pasting properties of starch were measured using a rapid visco-analyzer RVA-3 (FOSS Japan) at 7% (w/w) starch concentration. Values within a column followed by different letters are significantly different according to Tukey’s test (*P* = 0.05). *^a^* The whiteness of starch was measured by Powder Whiteness Tester C-130 (Kett).

**Table 5.** Evaluation of Michishizuku and control varieties for resistance to foot rot disease

Variety	2021					2022			
	Disease incidence*^a^* (%)	Yield of marketable storage roots*^b^* (kg/a)	Ratio of marketable storage roots*^c^* (%)	Resistance evaluation*^d^*		Disease incidence*^a^* (%)	Yield of marketable storage roots*^b^* (kg/a)	Ratio of marketable storage roots*^c^* (%)	Resistance evaluation*^d^*
Michishizuku	33 cd	147 b	51 b	moderately strong		29 cde	389 a	83 a	moderately strong
Koganesengan	90 a	24 c	16 c	weak		83 ab	125 bc	59 b	moderately weak
Shiroyutaka	33 cd	150 b	67 ab	moderately strong		64 b	126 bc	53 bc	moderately weak
Tamaakane	4 d	342 a	92 a	(strong)		3 e	431 a	98 a	(strong)
Konaishin	28 cd	272 a	68 ab	(moderately strong)		10 de	406 a	91 a	(moderately strong)
Benimasari	32 cd	97 bc	45 b	(moderately strong)		26 cde	96 bc	39 cd	(moderately strong)
Ayamurasaki	47 bc	78 bc	58 b	(medium)		33 cd	171 b	90 a	(medium)
Kokei No.14	75 ab	18 c	12 c	(moderately weak)		57 bc	65 c	43 bcd	(moderately weak)
Daichinoyume	100 a	10 c	10 c	(weak)		100 a	71 c	30 d	(weak)

Michishizuku and control varieties were grown in a field infested with foot rot for two years (2021 and 2022), and evaluated for resistance to foot rot. Tamaakane, Konaishin, Benimasari, Ayamurasaki, Kokei No.14, and Daichinoyume were examined as indicator varieties. The level of resistance to foot rot for each indicator variety is indicated in parentheses. The planting dates were May 6, 2021 and May 1, 2022. The harvest dates were October 13, 2021 and October 17, 2022. Disease incidence was examined on September 2, 2021 and October 7, 2022. Values within a column followed by different letters are significantly different according to Tukey’s test (*P* = 0.05). *^a^* Disease incidence = Percentage of plants rotted at the basal part of a stem. *^b^* Marketable storage roots were defined as those that showed no foot rot symptoms or field sprouting. *^c^* Ratio of marketable storage roots = Yield of marketable storage roots / Total yield of storage roots. *^d^* The resistance class to foot rot was evaluated based on the disease incidence, considering the marketable storage root yield and ratio.
